# Supplementary material for: The genome sequence of an ichneumonid wasp, Apaeleticus inimicus (Gravenhorst, 1820) (Hymenoptera: Ichneumonidae)
Source: Wellcome Open Res. 2025 Oct 6;10:550. [Version 1] doi: 10.12688/wellcomeopenres.24945.1 (PMC12640502; doi:10.12688/wellcomeopenres.24945.1)
Supplement: Peer review history

# Reviewer report: Reviewer response for version 1

**DOI:** 10.21956/wellcomeopenres.27477.r137393

**Published:** 2025-11-22

**Authors:** Nicola J Nadeau

**Affiliations:** 1 The University of Sheffield, Sheffield, UK

The work is thorough and conducted to the recognised standards in the field. The background is useful, although slightly on the short side. Is the species is haplodiploid or assumed to be haplodiploid? It would be useful to add this information to the background section, in order to explain the lack of sex chromosomes. It might also be useful to justify the choice of a diploid female for sequencing rather than a halpoid male, since presumably the latter would have allowed for cleaner sequencing of a single haplotype without any possibility for misassemblies or incorrect phasing of the haplotypes.

Are sufficient details of methods and materials provided to allow replication by others?

Yes

Is the rationale for creating the dataset(s) clearly described?

Yes

Are the datasets clearly presented in a useable and accessible format?

Yes

Are the protocols appropriate and is the work technically sound?

Yes

Reviewer Expertise:

evolutionary genetics/genomics, lepidoptera

I confirm that I have read this submission and believe that I have an appropriate level of expertise to confirm that it is of an acceptable scientific standard.

# Reviewer report: Reviewer response for version 1

**DOI:** 10.21956/wellcomeopenres.27477.r136636

**Published:** 2025-11-11

**Authors:** Ariel Chipman

**Affiliations:** 1 Hebrew University of Jerusalem School of Pharmacy, Jerusalem, Jerusalem District, Israel

The authors report own the genome of an ichneumon wasp Apaleticus inimicus, sequenced from an individual from England as part of the Darwin Tree of Life project. The sequencing and assembly were done according to standard protocols for this type of work. The manuscript is clearly written and the descriptions of the work are given in sufficient detail. I see no technical or conceptual problems that should prevent this manuscript from being indexing.

Are sufficient details of methods and materials provided to allow replication by others?

Yes

Is the rationale for creating the dataset(s) clearly described?

Yes

Are the datasets clearly presented in a useable and accessible format?

Yes

Are the protocols appropriate and is the work technically sound?

Yes

Reviewer Expertise:

Arthropod evolution.

I confirm that I have read this submission and believe that I have an appropriate level of expertise to confirm that it is of an acceptable scientific standard.
